# Psychiatric Experiments with “Community” Under Dictatorship and Authoritarianism: The Case of the Protected Commune Experience, 1980–1989

**DOI:** 10.1007/s11013-024-09868-2

**Published:** 2024-07-01

**Authors:** Cristian Montenegro

**Affiliations:** 1 Wellcome Centre for Cultures and Environments of Health, University of Exeter, Exeter, UK; 2 Escuela de Enfermería, Pontificia Universidad Católica de Chile, Macul, Chile; 3 Department of Global Health and Social Medicine, King’s College London, London, UK

**Keywords:** Psychiatric asylums, Dictatorship, Community-based psychiatry, Mental health reform, Oral history

## Abstract

In Chile, a long and oppressive military regime (1973–1990) dismantled emergent initiatives for the deinstitutionalisation of psychiatric care, imposing a neoliberal constitution that opened public services to market forces and limited the state's role in health and social care. After being associated with communism and socialism, community-based mental health work was banned, and socialist psychiatrists were silenced through torture or exile. However, some therapeutic initiatives persisted, such as the “Protected Commune” (PC) initiative within the El Peral psychiatric asylum. The PC attempted to mimic a real town inside the asylum's gated perimeter. It featured an ecumenical chapel, a school, and various “council” departments like recreation, education, waste, economy, and health. Paths received names, wards became districts, and patients and workers were assigned new, democratic roles, all while the authoritarian regime entirely controlled the “outside” world. The initiative ceased with the return of democracy in 1990. Deemed an eccentric and negligible episode, the PC is often seen as an interruption to the radical community-based experiences of the pre-dictatorial era. Drawing on archival research and oral history interviews with participants, this paper examines how the PC harnessed the notion of community to navigate the complex socio-political landscape of the dictatorship. Differing from established accounts of the political uses of psychiatry under authoritarianism, the study positions the PC as a prism for understanding the contradictory ways in which the idea of 'community' has been able to transcend radically opposed social and political regimes, becoming a core feature in the vocabulary of mental health reform, despite its ambiguities.

## Introduction

### The Origins of El Peral Hospital

El Peral Hospital is one of four single-purpose psychiatric hospitals in Chile and one of three that still provides long-term hospitalisation. Established in 1928 as an Open Door Colony^1^, it was designed to accommodate patients deemed beyond recovery in the National Mental Asylum (Manicomio Nacional) (Gómez & Villanueva, 2010). The hospital is situated on the outskirts of southeast Santiago, where the incline of the Andes hindered urban development for many decades^2^. It was eventually reached by accelerated suburban sprawling, beginning in the late 1980s^3^.

**Figure:**
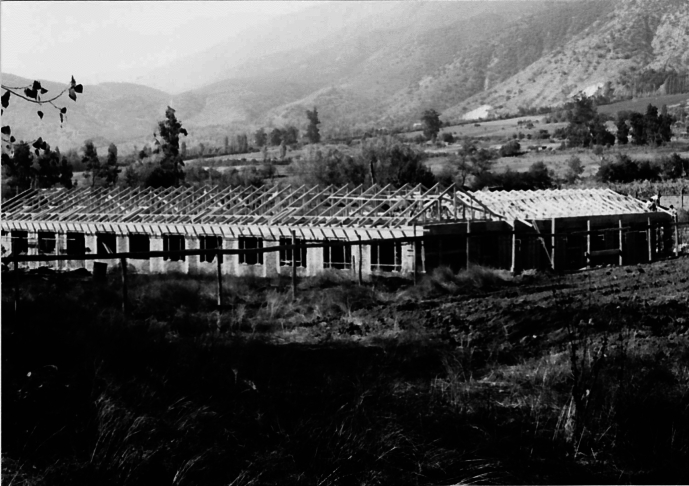
Image 1

**Figure:**
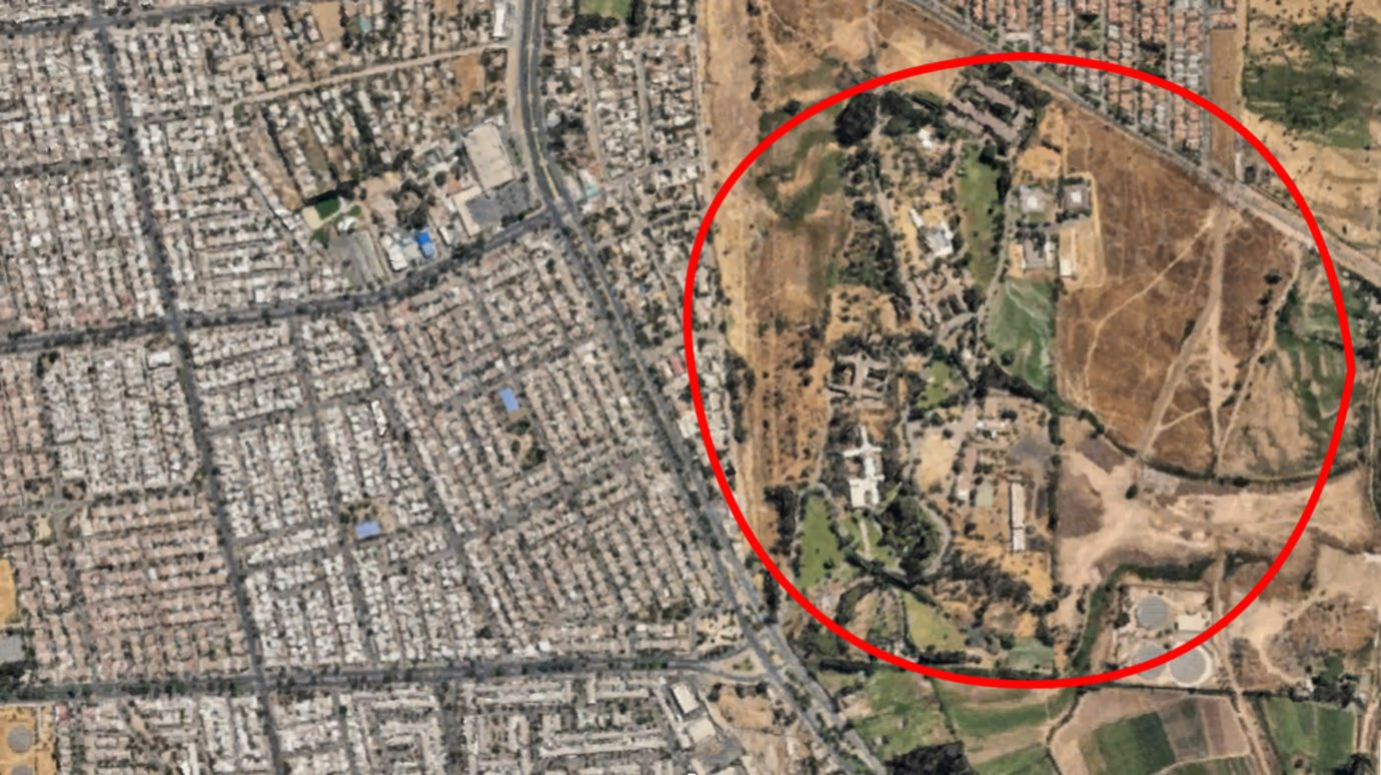
Image 2

The colony was founded to enable psychiatric patients to achieve self-sufficiency through agriculture, reflecting the prevailing belief in rural life's therapeutic and moral superiority over urban existence (Allmond, 2017). The colony occupied a substantial plot of land, or “fundo”, designated for farming and livestock. The first cohort of workers consisted of local peasants who cultivated the land and lived in a dedicated village within the fundo (Gómez & Villanueva, 2010).

Between 1967 and 1971, the closure of the Santiago Hospice led to the transfer of 200 patients, primarily those with neurological and physical disabilities but without psychiatric conditions. This transfer further exacerbated overcrowding at El Peral, contradicting its original vision of nature-based rehabilitation. Consequently, the colony's population exceeded 1000 patients (Gómez & Villanueva, 2010).

### Reformist Ambitions and Therapeutic Experiences Before and During the Dictatorship

Starting in the 1950s, alternative approaches to psychiatric care were explored throughout South America, coinciding with strong criticism of the abuse, misery, and abandonment experimented by patients. These approaches were influenced by Latin American Social Medicine, Preventive and Community Psychiatry in the USA, the Therapeutic Community approach in the United Kingdom, and Franco Basaglia's “Psichiatria Democratica” in Italy (Ablard, 2003; Amarante, 2022; de Almeida, 2020; Araya & Martínez, 2021; Kritsotaki et al., 2016; Molina et al., 2022).

In Chile, noteworthy among these initiatives was the “intra-community psychiatry” project developed by Juan Marconi between 1969 and 1970 in the south of Santiago (Marconi, 1972, 1973a). This project aimed to address the prevalent social and medical problem of alcoholism in the country, eventually expanding to encompass “neurosis” and child sensory deprivation. The program involved a structured hierarchy of training, support, and delegation of roles to local community representatives, who became service providers and co-creators of the strategy (Marconi, 1972). Marconi's vision for psychiatric reform was heavily influenced by the ascendancy of democratic socialism through the Unidad Popular coalition and the leadership of President and physician Salvador Allende (Montenegro, 2023).

The onset of a CIA-funded dictatorship in September 1973 abruptly halted these ambitions and plans. The military authorities, operating under the Cold War doctrine of National Security and the “internal enemy” thesis (Timmermann & Korstanje, 2016), perceived community-based mental health work as an extension of left-wing political organising and thus sought to suppress it. Furthermore, Marconi’s and others’ embracing of behaviouralist psychology—including the work of Ivan Pavlov—was wrongly seen as reflecting Soviet influence (Todes, 1995; Vera-Villarroel et al., 2006).

The dictatorship's impact on healthcare and mental health policy development was profound, disrupting a public health system established in 1952 that had significantly improved health indicators through an extensive network of care facilities (Horwitz et al., 1995). Prior attempts to implement community mental health initiatives, including pilot trials and public health programs, were shut down, and several key figures of the community mental health movement, including Marconi, were either exiled or sought refuge abroad, with others killed, jailed, or barred from public services. Psychiatry restricted its activities to universities and traditional medical spaces, including asylums. The only public mental health provision that endured the authoritarian backlash were ambulatory psychiatric services based in general hospitals and the treatment of alcohol dependence in primary care centres (Minoletti et al., 2012).

The regime intensified the ethical vacuum in which institutional psychiatry operated, characterised by a lack of legal accountability, independent advocacy, and media scrutiny towards the appalling treatment of patients. Political prosecution through violent raids became common across public institutions, including hospitals and psychiatric asylums, often with the direct support of hospital authorities (Escobar-Miguel, 2013; Ramirez, 2022). El Peral underwent intervention, with staff numbers slashed from 450 to fewer than 250 to manage over 1000 patients. This drastic reduction worsened the dire conditions of neglect (Gómez & Villanueva, 2010).

Upon returning to Chile in 1976, 3 years after the dictatorship began, Marconi continued his work under challenging conditions. With broader community-based initiatives now unfeasible, he proposed low-cost, targeted programs that could be implemented within psychiatric institutions (Araya & Leyton, 2021; Norambuena, 2017). This led to a collaboration between the University of Chile’s Department of Psychiatry and El Peral, aimed at transforming the institution into a therapeutic and rehabilitative space through the Token Economy Programme (TEP). Active from 1976 to 1978, the TEP was a comprehensive rehabilitation strategy for chronic patients. It combined behavioural learning techniques with a system of delegated tasks, designed to engage the entire asylum workforce in the rehabilitation process to socially reintegrate two-thirds of the 1200 children, women, and men housed (Araya & Leyton, 2021; Montesinos, 2018).

However, conflicts between the Hospital and the University led to the sudden termination of the TEP and other joint projects. Subsequently, a new director, appointed by military authorities, tried to salvage parts of the TEP. This effort led to the transformation of a section of the hospital’s chronic ward into what was later called the “Protected Commune” (PC)—an attempt to create a city-like environment within the asylum.

The “Protected Commune” (PC) experience is largely absent from narratives of psychiatric reform in the country. It is often seen as belonging to a lost period in the evolution of psychiatric services towards prevention and community-based care, an evolution interrupted by the dictatorship. This article, drawing on oral history interviews, demonstrates how the concept of “community”, as realised and practiced through the PC, serves as a crucial link between the ambitious political visions of 1970s psychiatry and the technocratic “community mental health” policies of the 1990s.

### Policy Paths After the End of the Dictatorship

By the end of the dictatorship, four mental hospitals consumed most of the countries’ mental health budget (Minoletti et al., 2010). The return of democratic institutions ignited a series of reforms guided by the ‘Conference for the Restructuring of Psychiatric Care in Latin America’ and its ‘Caracas Declaration’, which was signed by most countries in the region in 1990. Subsequently, a long overdue process of deinstitutionalisation and decentralisation began (Caldas de Almeida, 2005).

Two distinct policy paths followed the Declaration. Countries like Brazil and Argentina embraced significant reforms and local-level experimentation, whereas Chile focused on expanding services through community-based facilities (Maass et al., 2010). This approach is evident in three national mental health plans (1993, 2000, and 2017) that align with WHO’s technical recommendations, promoting the expansion of services through primary healthcare and initiating a gradual—and still incomplete—process of deinstitutionalisation (Minoletti et al., 2010).

For decades, international and national mental health authorities have advocated for transitioning from centralised, isolated institutions to community-based mental health services (Cayon, 2016; WHO, 2013). In these discussions, “community” often serves as an aspirational goal, marking the shift from traditional asylum-based care to modern mental health practices. Additionally, the drive to scale up services, a core aspect of contemporary Global Mental Health initiatives (Bayetti et al., 2023), has promoted an abstract concept of “community” as a generic geographical area where services are delivered. This article examines the variability and specificity with which “community” is conceptualised within mental health discourse, particularly in initiatives like the Protected Commune. It explores how the idea of community and its associated practices have been adapted to fit radically different socio-political contexts across times and regions (Antic et al., 2023).

This article is part of the “Recontextualizing Psychiatric Deinstitutionalization (Redepsiq)” project, whose goal is to trace and contextualise the practices and policies of psychiatric deinstitutionalisation that began in Chile in the early 1990s, looking at El Peral Hospital, a paradigmatic case of this complex transition in the country (Gómez, 2012). The article investigates the origins, goals, and main characteristics of the Protected Commune (PC), as well as the hopes and critiques it raised among both professional and non-professional workers. It problematises available reconstructions of mental health policy development in the country and the region, suggesting that the PC served to reformulate the scope of a central idea in the vocabulary of reform—both before and after dictatorship: The idea of “community”.

## Methods

Oral history interviews were conducted with a pool of forty-four individuals, between March 2020 and November 2022. The COVID-19 pandemic and consequent social distancing regulations significantly limited the potential for participant observation within the hospital environment. As a result, the study's findings primarily rely on the testimonies from interviews and the information from the available documents.

The interviewees encompassed present and former workers and authorities at El Peral, incumbent and past health and mental health authorities, international leaders involved in psychiatric reform processes linked to the Chilean experience, representatives from civil society, service users, and family caregivers.

Oral history is particularly valuable for including the perspectives of those often overlooked in traditional historical accounts, especially in oppressive settings like colonial slavery where dominant narratives prevail and marginalised voices are silenced (Thompson, 2017). However, we were unable to find any patients who participated in the Protected Commune (PC) during the 1980s. Representatives responsible for patient discharges and managers of community residential services indicated that most of these patients had either passed away or were too frail to participate in interviews. Additionally, COVID-related social distancing measures during our research further restricted our access to potential interviewees.

Despite this crucial absence and considering the prevailing dismissal of the Protected Commune as irrelevant or valueless, this study contributes a richer, albeit still incomplete, perspective to the social history of mental health policy in Chile and South America. Importantly, it underscores the need for future research to actively seek and incorporate the experiences of the inmates and users of El Peral and similar institutions.

Transcriptions of the interviews were imported into Atlas.TI, a qualitative software package, to assist with the analysis. A thematic analysis approach was applied, utilising a coding framework combining deductive and inductive logic. The inductive logic linked the generation of codes to the overarching goals of the Redepsiq project. In contrast, the deductive logic facilitated the identification of emerging themes and unexpected insights from the data.

Conducting and interpreting oral history interviews involves methodological and ethical challenges, such as interviewer bias and informant reliability (Kirby, 2008). People often remember and describe past events in ways that align with their personal viewpoints or interests. As Halbmayr (2009) points out, memories are dynamic; they continuously evolve as new experiences overlay older ones, allowing past events to fade or be reinterpreted with each recollection. Recognising these complexities, this paper does not seek to provide a definitive account of what transpired within the Protected Commune or El Peral. Instead through a diverse collection of first-person accounts, considers how, under authoritarian conditions, a specific view of community mental health emerged.

## Findings

### “We had to put some limits on what psychologists were doing”. The Demise of the Token Economy Programme and the Origins of the Protected Commune

Psychologists started to complain and finally, they left, right? And then, the occupational therapists and nurses were left trying to keep the rehabilitation programs afloat.

Alejandro Riveros, who worked at El Peral between 1978 and 2000, began his career as a nurse at the hospital. The hospital was severely understaffed during his initial years, with a small group of nurses catering to approximately 3500 patients. He tells grim tales of patients lying in their faeces, succumbing to hunger, infectious diseases, or freezing to death. Back then, the primary role of nurses in the hospital was, starkly put, to keep patients alive.

Alejandro was one of the ten nurses assigned to various wards in the hospital. His first assignment was in Ward 7 (W7), a place reserved for long-term patients with complex behavioural issues and low responsiveness to treatment. Many professionals depicted the ward as a sombre, pestilent dungeon.

Alejandro and other nurses began their duties just as the Token Economy Programme (TEP) was starting to collapse. The TEP is the most immediate and relevant context to understand the origins and nature of the Protected Commune.

The TEP was initially implemented in Ward 10 (W10), where patients with the lowest behavioural and social skills were housed. The program started with a cohort of thirty-one patients, eventually expanding to include all 150 patients in the ward. The programme consisted of a highly structured system of behavioural conditioning, demanding continuous monitoring of patients’ daily activities. A list of adaptive behaviours considered crucial for life outside the hospital was established. These included personal hygiene, food handling, basic social interaction, and money management, among others. A dedicated team of psychologists monitored and reinforced these behaviours while trained nurses and other workers supervised the program outside of working hours. According to testimonies, 17 patients progressed to a stage where they could be “externalised”. Still, the lack of stable support outside the asylum often challenged this option (Araya & Leyton, 2021).

Inside the hospital, a separate area was allocated for TEP participants, which included bedrooms and dining rooms that served as immediate reinforcement and observation areas. Additionally, a simulated “store” was established to teach essential money management skills crucial for life outside the hospital (Araya & Leyton, 2021).

Alejandro Riveros was one of the nurses trained to implement the TEP. In his view, the approach went to extremes that, from a nursing perspective, were challenging to execute and conflicted with basic care considerations.

Sometimes we had to draw a line on what the psychologists were doing. We had disagreements with them because, you know, we were located at the foothills, and it frequently snowed, dropping to two or three degrees below zero during winter. The psychologists insisted that toilet training had to be continuous. Patients used electric mattresses, and a buzzer would go off when a patient wet the bed. We had to wake them up immediately, get them out of bed, remove the wet sheets, and take them to the bathroom to wash them with cold water as punishment so that they wouldn't do it again. This led to respiratory issues, pneumonia, and—I'm not even exaggerating—deaths from respiratory problems. We couldn't stand for that.

According to Riveros, the strict operant conditioning carried out by the psychologists was impractical and hazardous, given the locale and the nature of the facilities. For other non-professional workers, the initiative disrupted long-standing routines based on the prevailing notion that patients could not recover. The idea of rehabilitation for social inclusion was barely conceivable under this viewpoint. Angélica Contreras, who worked as a secretary in the hospital from 1973 to 2017, corroborates this perspective:

When the token economy came about, which was in 1978, at first, there was resistance, especially from the older workers [gente antigua], because it was an implementation that wasn’t consistent with the routine that was carried out for decades. The older workers believed that the situation for patients was unchangeable, thinking, “This is black, and it will be black until the end” [referring to patients' condition and status].

Apart from worker criticisms and resistance, the initiative started disintegrating due to a combination of internal tensions and political pressures. As the 1970s drew to a close, hostilities and control intensified under the dictatorship, leading to increased scrutiny and interference in mental health initiatives perceived as ideologically contentious. By the end of 1978, it became evident that the TEP could no longer operate under such conditions (Araya & Leyton, 2021). According to Marconi, the dictatorship became suspicious and abruptly halted the programme, citing ideological and political reasons rather than technical failures (Mendive, 2004). From 1978, he started receiving threatening communications, which culminated in 1979 with the intervention of the Department of Psychiatry and Mental Health at the University of Chile, which led to the dismantling of the TEP public disqualifications, transfers, and threats of relegation to other institutions (Araya & Leyton, 2021).

In 1980, following Marconi's resignation from his position at the University of Chile, a temporary director was appointed who halted most rehabilitation programs at El Peral. The professional staff, trained in behavioural analysis and modification, resigned en masse, leading to the collapse of the programmatic core (Araya & Leyton, 2021). This disintegration severed all connections with the hospital's bureaucracy, eliminating any possibility of follow-up. The patients were left in a state of neglect, accumulating behavioural points that could no longer be exchanged for any tangible rewards, exacerbating their conditions (Montesinos, 2018).

Between 1980 and 1981, without a formal announcement, the TEP project ended, thereby closing this short experimental phase. Besides notable exceptions (Morales Sáez, 2023), existing historical accounts of El Peral typically assume that after the end of the TEP, there are no further developments and resume with the end of the dictatorship in 1990.

### “A small city, like any other city in Chile”. Characteristics of the Protected Commune

Dr Cristian Wulf, the new director of El Peral, paediatrician, and psychiatrist, had experience working with children with developmental disabilities. One of his first actions in the asylum was to bring in “special education” practitioners to work with approximately 50 underaged residents. Margarita Martinez, one of these practitioners, recalls:

Dr. Wulf introduced young professionals into the hospital gradually: occupational therapists, special educators, social workers, etc. He initiated a movement founded on Maslow's Hierarchy of Needs, urging us all to prioritise the basic needs of the patients: food, clothing, space, and the like. This was critical because, back in the '70s, people frequently died there.

Besides focussing on patients' basic living conditions, Wulf's most notable and contentious legacy was the Protected Commune (PC).

Dr. Wulf believed that this group of 3,000 patients living on the hill was like a village or even a small city, like any other city in Chile. He reasoned that since this was a permanent population, why not provide them with an organisation like a small commune? So, he appointed a mayor, and guess who that was? It was me. (AR)

Alejandro Riveros provides further details about the PC. It encompassed a waste, order, and decoration department responsible for maintaining the facilities. The “wards” were rebranded as “districts” (Unidad Vecinal), each given a new name, and the corresponding buildings were painted in various colours. Patients' clothes corresponded to these colours. Passageways connecting the wards became streets with names. A central space between the buildings was transformed into a town centre, and a specially built ecumenical chapel was established for religious celebrations.

A sports and recreation department organised events like an annual “Mental Health Olympics” and a “holiday exchange” program for patients. For the latter…

Buses were hired using money from the patients, managed by the welfare department, to transport 40 of our patients to El Salvador Hospital in Valparaiso^4^. The team there took them to the beach and other areas for around 10 days. Afterwards, 40 patients visited El Peral and stayed here, and we took them to explore the underground trains and other parts of Santiago.

The same department organised biweekly dances, turning the largest ward (number 7) into a dance floor that could accommodate around 300 people.

Orchestras were hired, playing cumbia and rancheras, and then sandwiches, hard-boiled eggs and powdered juice were prepared… and then from 7 to 10 at night, there was a dance. Some patients were brought, those with a good level and able to join this type of activity.

The work department, led by occupational therapists, managed various workshops, including a broom and rug factory. Like any other city, some form of medical care was available, as explained by Betty Gomez, a nurse and Riveros' colleague at the time:

Patients were dying from cold or hunger when we first came here [late 70s]. So, the first thing we did was to check their pulse and pressure to see if they were alive. With the protected commune, we introduced a primary healthcare model within the hospital. That meant monthly health checks for people, including weight, blood pressure, temperature, food, clothing, and all their basic needs.

This corroborates the foundational vision of the PC: Rather than being a space for medical treatment, the hospital had to function as a small city permanently housing a stable population.

The PC also had a council with representatives, including directors of each department and managers of the different wards, now renamed “districts”. Riveros mentioned that “some patients with better behavioural levels” were invited, along with the few family representatives who maintained contact with the patients. Notably, the council operated as a parallel hierarchical structure within the hospital, assigning new roles to non-professional staff.

### “Because workers react to how bosses treat them”: The Situation of Non-professional Workers in the Commune

According to various interviewees, the primary objective of the PC was to establish an environment that reshaped the experience and situation of patients *and* workers. The PC addressed the contentious relationships between professional and non-professional workers, such as administrative assistants (secretaries), nursing assistants, cleaning staff, and others. The conflict between professionals and “paraprofessionals”, present across times and regions (Smith, 2023), can be traced back to the origins of the asylum. Mario Villanueva, a social worker at the hospital during the 1990s and a historian of El Peral, explains:

At the beginning of El Peral (…) there weren't enough workers. As a result, the farmers who worked on the surrounding lands were hired. Over time, jobs began to be passed down from father to son. Not only that, but these workers eventually married each other, and this tightly-knit group ultimately took control of the hospital. This was especially true in the past when doctors came to work their five or eight-hour shifts, and patients spent most of their time with these workers, workers who managed, cared for and, very often, abused these patients. So, when professionals arrived and attempted to implement changes, they faced fierce, closed-off opposition from workers.

The low-intensity, passive nature of the PC accommodated workers' reluctance to engage in treatment or rehabilitation processes. Moreover, they valued the initiative, feeling acknowledged and supported by the hospital authorities. As Waldo Contreras, a nursing assistant since 1980, explains:

Cristian Wulff, I believe, led one of the best administrations in the hospital's history. During that time, he established the Protected Commune. He was genuinely concerned with the way people worked. If he saw you, he'd say, "Hi Waldo, how are you? How's your wife? How are the patients? Do you need anything in your ward?" I'm not lying; some workers had no money or food for their kids... because of the dictatorship. And this administration would give you money, bring milk and food to the people, and we responded well... because workers react to how bosses treat them.

The PC not only accommodated the way people worked but also created an alternative governance structure that empowered non-professional staff headed by the mayor and its council. The administration of each district was entrusted to non-professional workers reporting directly to the mayor.

Within the logic of the commune, an administrative chief was appointed for each ward. It was generally a secretary, and this new level of authority generated conflicts with other workers, such as nurses. (Betty Gómez).

In the early 1980s, the repressive regime profoundly shaped social life outside the asylum. A State of Emergency has been renewed continuously every 6 months since 1973 to prevent demonstrations. Systematic torture, disappearances, and other forms of terror dismantled grassroots political organising, tearing apart collective life. The city surrounding the asylum was a minefield (Eltit, 1997), devoid of normal, spontaneous interaction, without a “community” for patients to be discharged.

No discharge meant no rehabilitation, no stages to be achieved, no changes to be measured, and no anticipation of an independent life outside. The most that could be done for “chronic” patients was to provide a basic level of well-being within the asylum.

In this hostile and closed context, the commune—and El Peral—became a small oasis for these workers. They hold memories of parties, celebrations, and more organic and spontaneous interactions with patients, unfettered by the division of labour and the accountability required to support rehabilitation aimed at externalisation. However, other testimonies present a different perspective.

### “It was like a fantasy, a Romantic Thing”: Criticisms of the PC

El Peral was Claudia Carniglia’s first job as an Occupational Therapist. She started in 1983 and stayed there for 15 years. She became part of the recently created Occupational Therapy Service, a rehabilitation area holding workshops for patients with the potential for independent living. When talking about the PC, she, like others, emphasises the “intramural” nature of the experience.

There was a director who implemented this model of organisation, the protected commune. What was previously Ward 7 was now called "District 7". There were Districts 4 and 5 and so on. There was a mayor, a Sports and Recreation department, a Work department... but everything was inside this citadel; that was the logic.

Margarita Martinez confirms this, introducing an ambivalence between enthusiasm and distance:

It was all inside… it was all inside, we did what we could, and we were content, young people accomplishing things… but with the distance of time, one says, ‘sure, we did things for the sake of patients, but it was all inside’.

Mauricio Gomez, a psychiatrist at the hospital during the 1980s and a central figure in its transformation during the 1990s and 2000s, radicalises this observation.

It was a very curious thing... the wards became districts, and the medics worked through a sort of polyclinic, almost like domiciliary care. It was like a fantasy, a sort of romantic thing but, for me, it was a lie, all fake because, simultaneously, all the serious behavioural issues - and there were a lot of those - were dealt with brutal coercive measures: cages, cells, padlocks everywhere. There was a yard for the "gatosos”^5^, those with severe mental retardation, people who wet themselves, soiled themselves, who were like... lying on the ground all day; it was horrendous.

The whole initiative only considered a small proportion of chronic patients. Those groups with complex behavioural problems or neurological conditions were subjected to a double exclusion: isolated from society and excluded from the commune inside the asylum. Traditional routines of neglect and abuse carried on.

When I first asked her about the PC, Andrea Bahamondes, who started training as a psychiatrist in El Peral in 1981, replied:

I cannot tell you that I have perceived anything other than large, dark, poorly ventilated buildings with a highly undesirable odour, overcrowded with patients sleeping in beds less than two metres apart.

Dr. Bahamondes' perspective on the PC differs from that of most other interviewees. She studied medicine at the Universidad de Chile right after the start of the dictatorship. As a medical student, she was an active organiser, enduring political prosecution during the dictatorship regime. She was deeply aware of the anti-institutional tendencies in international psychiatry—particularly in the UK—and saw it as her challenge to transform El Peral into a space for legitimate, professional medical care. Speaking about her first steps in the hospital, she comments:

I knew that this was going to be an event that was going to mark my life… it was a dictatorship, it was the way to also channel my personal stance regarding what was happening in Chile, in an area where I was also driven by my medical vocation.

Her view of the PC is consistent with this political and professional call. For her, the problem with the PC goes beyond its internal, selective, or superficial character, extending to the origins of the experience:

At one point, all the patients living in the "chronic" section, given their definitive disability, were granted a disability pension by the State. By creating a "commune", the director - through the "mayor" - could act as a legal representative for these people, withholding their pensions, which was their money and which they could have used for their interests or given to their families. They didn't have to pay for the hospital! The State had the obligation to provide the hospital. But the authorities devised all kinds of strategies to save budgets for the nation, taking advantage of the patient's situation, and that was very scandalous (...) So, the protected commune was a kind of setup to gather that money, and with that, you could hire some assistants, buy deodorants, paint walls, and make it appear as if you were catering for the patients' needs, but it was their own money!

And she continues.

So you couldn't receive your pension anymore, but the commune provided you with food, a shirt (the same for everyone), a bed (with a sheet and two blankets), one cologne, and one deodorant a month... And as it usually happens in these institutions, a bus was rented at some point, and a group went somewhere and came back... There was a Christmas celebration that, obviously, was only for the workers and the professionals, and the patients were locked up elsewhere. I don't see where the money could have come from if it wasn't from the protected commune (...) That design remained untouched until the return of democracy. That was the legacy of the protected commune.

Dictatorship led to massive unemployment and poverty (Bresnahan, 2003). Underfinanced public institutions developed various strategies to deal with the intentional public shortfall created by the regime, whose goal was the swift implementation of neoliberalism. Within psychiatric institutions, the main resource to draw on was the patients themselves, using their money to provide for their basic needs. The PC provided the legal scaffolding for this extraction. Dr Marin Cordero, a key figure in the struggle for human rights in the mental health field in Chile, made a broader point about the situation of El Peral and other psychiatric institutions in history, commenting:

The following principle is behind these houses of horror [referring to psychiatric asylums]: if they [patients] do nothing, then you do nothing for them. If you don't provide basic living conditions for these persons, you will attribute their situation to themselves, to their passivity, and that creates a vicious circle.

### “This ceased to be seen as natural”: The End of the Protected Commune

I don’t remember exactly… I don’t know for how long after 1990 Wulff was the director, maybe a year or so. After that, Haydee Lopez assumed as director, and that led to significant transformations. Andrea Bahamondes was also part of that, and Martín Cordero played an important role too. At the same time, we had the first National Mental Health Plan [1993], we had the Caracas Conference [1990], and there was this deep questioning regarding the psychiatric hospital, especially the long-term “chronic” section. The main target of interrogation was this group of people who didn't recover, who stayed there and spent their lives in the Protected Commune. This situation ceased to be seen as natural or acceptable.

Claudia Carniglia worked at El Peral from 1983 to 1998. She later contributed to establishing a psychosocial rehabilitation area in a psychiatric unit at Hospital Barros Luco, a large public hospital. Subsequently, she held a coordinating position at the South-Metropolitan Health Service, which supervises the clinical and administrative operations of the health network across a vast area of South-Santiago.

Carniglia's perspective is shaped by her trajectory, which began inside El Peral and then moved into the integration of mental healthcare into the general health network and the development of residential alternatives for discharged asylum patients. Her quote highlights significant milestones in mental health, including the Caracas Declaration. This declaration was signed in 1990 by Latin American health authorities, including Chile. Signatories agreed that mental hospitals must cease being the centre of psychiatric care (Bolis, 2002; Levav et al., 1994). The 1993 National Mental Health Policy and Plan echoed and reinforced these principles (MINSAL, 1993). While life in El Peral in many ways remained unchanged after these initial post-dictatorship plans and declarations, testimonies and available written accounts confirm that the PC was no longer an acceptable management, treatment, and rehabilitation strategy and was officially terminated in 1990.

## Discussion

The return of democratic institutions re-started the transition towards a model of community-based services, as imagined by pioneers before the dictatorship (Marconi, 1999; Minoletti, 2016). In 1993, Chile's first national mental health plan formalised this shift, advocating a transition from traditional asylums towards integrated healthcare networks (MINSAL, 1993). Since then, most of what is written about mental health policy embraces a strong distinction between the past—symbolised by the asylum—and the future—represented by a decentralised network of community services and supports.

**Where does the Protected Commune fit within this historic narrative?** Aside from a few recent exceptions (Morales Sáez, 2023), the Protected Commune is seldom discussed and, when it is, it is often depicted as a minor, pseudo-therapeutic detour (Gómez & Villanueva, 2010)—a relic from a darker era, destined to end upon the return of democracy, when the project of a community-based mental health system could be resumed, and when actual communities could become the destination of both patients and services.

The dismissal of the PC in policy reconstructions can also be seen as a way to take distance from the dictatorship and its authoritarian legacy. Using Linz’s classic definition of authoritarianism (2000), the political context directly influenced what occurred inside El Peral: limited political pluralism and constraints on political parties and interest groups curtailed accountability and prevented any form of denunciation or advocacy for patients’ rights. The lack of a functioning public sphere and political interference in academic spaces hindered the discussion and development of alternative mental health approaches. This situation was exacerbated by the precarity of public services, enforced by the regime.

The interviews, nonetheless, reveal that the Protected Commune was not merely a manipulation of psychiatric discourses and tools to serve the dictatorship’s objectives, a trend observed in other countries under authoritarian regimes (Buoli & Giannuli, 2017). Instead, it was a survival strategy in front of a hostile political and social environment. A strategy that intensified and exploited the neglect that patients experienced before the dictatorship, in the absence of public oversight and control, and with diminished resources and staffing.

With the return of democracy, policymakers framed the dictatorship as a wasted period breaking the path towards progress in mental health policy, a path opened in the 1960s and 1970s by Marconi and others (Armijo, 2010; Minoletti, 2016; Minoletti et al., 2012). Contrary to this narrative, this section will argue that the Protected Commune (PC) actually served as an unlikely link between the innovative ideas of the 1970s and the post-dictatorial, internationally influenced vision of community mental health. Drawing on the testimonies explored and organised in this piece, the experience will be used as a lens to explore how psychiatry has imagined the idea of “community” and implemented some of its features. This involves rooting the PC experience in the sequence that goes from the revolutionary ambitions of the 1960s and 1970s through to the Token Economic Programme inside El Peral. We will argue that the Protected Commune serves as a critical moment of innovation and re-elaboration of the practice and the idea of “community” under the adverse social and political circumstances brought by the dictatorship.

### The Semantic Evolution of “Community” in Mental Health: From Revolutionary Visions to Enclosed Adaptations Under Dictatorship

In 1973, months before the coup d’état, Marconi published a paper called “The Chilean Cultural Revolution in Mental Health Programs” (Marconi, 1973b). The piece outlines a proposal for the integration of mental health systems development and the socialist revolution taking place in the country and the region. Grounding his ideas in historical materialism, Marconi proposed a final, “community” stage in psychiatry. This stage resulted from the epidemiological magnitude of mental health problems, which exceeded psychiatry's asylum-based capacities. He argued for transferring psychiatric services, education, and research directly to communities, just as other industries in a context of nationalisation (Marconi, 1973a, 1973b; REDACTED).

In the highly politicised context of a democratic socialist transition, “community” was represented as a collective force gradually controlling its destiny by and for itself. Mental Health Services Reform consisted of opening psychiatry to the political direction and the multiplying forces of the masses. “Community” was the engine and the steering wheel of this transformation. Simultaneously it was a “medium” for the multiplication of structurally precarious resources, including psychiatric treatments.

The dictatorship that followed dismantled Marconi’s revolutionary agenda, but the underlying needs and disparities in psychiatric care persisted. In 1976, the asylum became the last resort for implementing Marconi's ideas, albeit in a significantly altered form. This gave rise to the Token Economy Programme (TEP) and subsequently, the Protected Commune (PC), experiences that constitute moments of adaptation and re-elaboration of the practice and the idea of “community” under increasingly adverse social and political conditions.

The TEP gave inmates enough abilities to live *in* the community. While not a politically driven collective transforming society, community was, at the very least, the geographic endpoint of a trajectory of rehabilitation that started inside the asylum. What happens when this endpoint is blocked, and the geographical and relational space surrounding the asylum is made unrecognisable by years of curfews and relentless terror? In other words, how can psychiatry imagine and implement “community” under threatening and alienating conditions?

As suggested by Ramos in his study of the relationship between authoritarianism, psychoanalysis and community mental health in Argentina (Ramos, 2013), there is not a one-to-one correspondence between psychiatric theories and particular political positions. This implies that concepts like “community” undergo a form of semantic adaptation, where certain features are emphasised or minimised to align with the ideological needs and constraints of the prevailing context. The PC, in particular, showcases how three facets of “community” were selectively reinterpreted: protection, unity, and resource multiplication.

First, the PC served as a protective barrier against a hostile outside world. Isolated from the oppressive urban environment—the epicentre of repression—and mostly overlooked by authorities, the asylum effectively became a space where a sense of community could emerge. This is confirmed by the nostalgia expressed by workers, particularly those with deep ties to the asylum’s historical agricultural past.

Similarly, the PC helped El Peral authorities administer different forces and positions within the hospital, fostering unity and smoothing over the frequent tensions that clinical and rehabilitative initiatives like the TEP often exacerbated (Araya & Leyton, 2021). Instead of disrupting the deep-rooted affective networks among non-professional workers, the PC harnessed their loyalty to each other and to a static vision of the asylum. Specifically, it legitimised and structured informal alliances by establishing a new hierarchy and roles that complemented the formal hospital administration.

Finally, the PC tapped into the multiplying capabilities Marconi already associated with “community” in its project for a cultural revolution. The expensive and burdensome professional infrastructure required by the TEP could be diluted and retained. Instead of an intense regime of specialised rehabilitation activities premised on the possibility of externalisation, the PC turned the hospital into *the* destination. This shift rendered rehabilitation, assessment, and training superfluous. Furthermore, users’ sole resources—their pensions—served to offset the complete lack of material investment. Community was equivalent to enforced collective self-sufficiency in a context of scarcity and with no consideration for patients' preferences or rights.

“Community” is a multifaceted and controversial construct that has stirred considerable debate in mental health policy and practice (Elias et al., 2021; Hunter & Riger, 1986; Krause & Montenegro, 2017). For decades, at the level of policy discourse, it has stood as an aspirational target in the transition from asylum-based to modern mental health services. However, alongside these idealised visions, critics have argued that community-based mental health policies have been driven by neoliberal—and colonial (Quarshie, 2022)—agendas that aim to minimise state expenditure, exposing service users to fragmented and precarious support systems (Grob, 1995, 2014).

The concept of ‘community’ in mental health, much like Benedict Anderson’s ‘Imagined Communities’ (Anderson, 2006), is not just a setting but a socially and politically constructed idea that serves specific purposes and agendas. A focus on how community is imagined allows us to suspend the weight of the dichotomy between asylum-based and community-based approaches (Alarcón-Guzmán & Castillo-Martell, 2020), interventions (Castillo et al., 2019), and settings (Farmakas et al., 2014). Instead, it is possible to focus on how psychiatric experiments with ‘community’ have evolved practically and conceptually in response to broader social and political shifts. Such a focus could enrich our understanding of mental health policy across different cultural and temporal contexts, contributing to a global history of its foundational concepts (Antic et al., 2023)

In front of recurrent calls for transforming mental health systems in the image of communities (Mathias et al., 2024), the radical potentials developed worldwide and lost in the semantic evolution of the concept (Luhmann, 1995; Stäheli, 1997) constitute an underexplored source of relevant models of what can be done—and what should be avoided—in the name of community. Rather than dismissing the Protected Commune as a discontinuity in an otherwise logical progress towards modern, community-based mental health systems, the experience provides a prism to understand—and situate—the complex ideological and political affinities of “community” (Rose, 2000).

## Conclusion

This paper has explored the paradoxical existence of the “Protected Commune” (PC) within Chile's El Peral psychiatric asylum during the oppressive military regime of 1973–1990. This period saw the dismantling of initiatives for deinstitutionalisation, the banning of community-based mental health approaches, and the silencing of revolutionary psychiatrists through torture and exile. In the middle of this authoritarian silencing, the PC emerged as a unique attempt at transforming El Peral Psychiatric Asylum into an isolated microcosm mimicking a real town, with new relationships running parallel to the traditional hospital hierarchies to simulate the life of an “outside” that was not there anymore.

Mostly framed as an eccentric and discontinuous episode outside the official history of progress and modernisation of the Chilean mental health system, the PC merits renewed scrutiny. Its existence is not simply an interruption to the progress of community-based approaches but serves to understand the kind of psychiatric care dictatorship allowed and supported. Crucially, the kind of “community” that psychiatry was allowed to imagine.

The PC serves as a prism for understanding the ways in which ‘community’ can serve as a guiding horizon and a therapeutic practice across vastly differing ideological and socio-political milieus. This paper calls for the expanded study of how psychiatry is shaped by authoritarianism across different settings and temporalities and how ideas of “community” can adapt and persist across political regimes and social contexts.

## Footnotes

1 The term “Open Door Colony” refers to a specific kind of therapeutic community within the psychiatric care system, designed to provide a less restrictive treatment environment than traditional asylums. Patients in these settings often have more freedom to move around and are encouraged to engage in occupational therapy, often based on agricultural or crafts-related work. For more details, see Wright, 1997.

2 Image 1 is a picture taken in 1992 by British psychiatrist Tom Harrison.

3 Image 2 is a recent aerial view of El Peral complex.

4 A psychiatric asylum located in the coastal area of Valparaiso, located 140 km away from El Peral.

5 From the French *gâter* (“to spoil (by soiling oneself with feces)”) + *-eux* (‘Gâteux’, 2023)
